# Social interventions to support people with disability: A systematic review of economic evaluation studies

**DOI:** 10.1371/journal.pone.0278930

**Published:** 2023-01-20

**Authors:** Bernice Hua Ma, Samia Badji, Dennis Petrie, Gwynnyth Llewellyn, Gang Chen

**Affiliations:** 1 Centre for Health Economics, Monash University, Caulfield East, Victoria, Australia; 2 Centre for Research Excellence in Disability and Health, Parkville, Victoria, Australia; 3 Centre for Disability Research and Policy, The University of Sydney, Sydney, New South Wales, Australia; University of Wyoming College of Health Sciences, UNITED STATES

## Abstract

Social interventions are essential in supporting the health and well-being of people with disability, but there is a critical need to prioritise resources for those that provide the best value for money. Economic evaluation is a widely used tool to assist priority setting when resources are scarce. However, the scope and consistency of economic evaluation evidence for disability social services are unclear, making it hard to compare across interventions to guide funding decisions. This systematic review aims to summarise the current evidence in the economic evaluation of social services for people with disability and to critically compare the methodologies used in conducting the economic evaluations with a focus on the outcomes and costs. We searched seven databases for relevant studies published from January 2005 to October 2021. Data were extracted on study characteristics such as costs, outcomes, perspectives, time horizons and intervention types. Overall, economic evaluation evidence of social services for people with disability was scarce. Twenty-four economic evaluations were included, with the majority conducting a cost-effectiveness analysis (n = 16). Most interventions focused on employment (n = 10), followed by community support and independent living (n = 6). Around 40% of the studies addressed people with mental illnesses (n = 10). The evidence was mixed on whether the interventions were cost-effective but the methods used were highly variable, which made comparisons across studies very difficult. More economic evidence on the value of interventions is needed as well as a more standardised and transparent approach for future research.

## Introduction

Social interventions are essential in supporting people with disability to fully participate in society [1]. However, they often face multiple barriers that hinder their full and effective participation. For example, inaccessible environments such as poor-designed sidewalks, institutional barriers such as inaccessible public transportation, communication barriers such as no plain language version of materials or attitudinal barriers such as discrimination. Social interventions supporting daily activities and other life aspects of people with disability (e.g., cooking, cleaning, transportation, employment, education) and broad policy interventions such as anti-stigma campaigns are thus critical to remove the barriers in society to allow people with disability to live full and equal lives.

Countries from the Organisation for Economic Co-operation and Development (OECD), on average, spend 2% of their Gross Domestic Product supporting people with incapacity, defined as spending due to sickness, disability and occupational injury [2]. However, the perceived unmet needs of people with disability continues to grow [3]. With continuously growing demand for disability support but a limited budget, governments need to decide what to fund and for whom. Economic evaluation is a valuable tool for determining the cost-effectiveness of interventions so that the value for money of alternative support options can be compared. An economic evaluation such as the cost-utility analysis, allowing comparisons across different studies for health interventions, is recommended by many government funding bodies such as the National Institute for Health and Care Excellence in the UK [4, 5] and the Pharmaceutical Benefits Advisory Committee in Australia [6].

Drummond et al. [7] and Weatherly et al. [8] summarised some common challenges in economic evaluations about a decade ago. The challenges include omissions of critical costs and benefits [7, 8], difficulties in measuring some costs and outcomes (e.g., measuring informal care) [8] and capturing the long-term costs and effects [7]. These challenges may result in difficulties in generating economic evaluation evidence that is comparable across studies. Economic evaluations of social interventions for people with disability may be particularly challenging because its impacts may stretch across the boundaries of health and social care, with cost implications for many different payers [9]. In addition, the need for interventions is likely to be more long-term, which is in line with disability typically being understood as conditions lasting longer than six months [10]. While we know the challenges, this systematic review examines whether the economic evaluations of social interventions for people with disability have overcome these challenges and moved forward. In particular, whether or not the current economic evaluation evidence is likely to be comparable such that it can assist funding decisions, and if not what we can do to improve the comparability of future research.

Specifically, the objective of this systematic review is to summarise the current evidence and critically assess the methods employed in the economic evaluation studies of social interventions for people with disability, with a particular focus on the costs and outcomes. It is anticipated that the findings from this review will provide a picture of the quantity and comparability of economic evaluation evidence in social interventions for people with disability, and guide further research in this field.

The paper is organised as follows: The next section explains the methods of conducting the systematic review, including the inclusion criteria, search strategy and extraction methods. We then present the results by firstly describing the scope of the studies, then presenting specific results about the costs and outcomes. We discuss the implications of the results and provides recommendations for future research in the Discussion section, and the last section concludes.

## Methods

The systematic review has been registered with the International Prospective Register of Systematic Reviews (PROSPERO registration number: CRD42020139498).

### Inclusion criteria

Studies published in English were included if they met the following three criteria.

1. The studies were economic evaluations, including cost-effectiveness analysis, cost-utility analysis, cost-benefit analysis and cost-consequence analysis. They all look at costs in monetary terms and differ in how the outcomes of the interventions are measured and compared. Cost-effectiveness analysis compares the benefits (for one specific outcome) relative to the costs in alternative interventions [11]. The outcome selected could be narrow such as full-time employment rates or may be broad (such as number of life years gained). Similar to cost-effectiveness analysis, cost utility analysis compares the benefits relative to the costs, however, it translates the health benefits into a common metric quality-adjusted life years (QALYs) such that the value for money of different interventions can be easily compared. The results are shown in forms such as cost per QALY gained. Cost-benefit analysis, on the other hand, provides net benefits of an intervention (benefits minus costs) by converting all the outcomes into monetary terms (e.g., an additional working day is worth $350). Therefore, if an intervention costs $5,000 and increases two working days, the net benefits calculated in the cost-benefit analysis is $4,300. Cost-consequence analysis considers the impact on a broad range of outcomes but does not attempt to combine outcomes into a single metric, and simply presents the changes in costs and outcomes separately without comparing them relative to each other (e.g., costs = $5000, benefits = 1 life saved & 5% higher employment rate).
2. The study only included social interventions which occurred outside healthcare settings and were carried out by individuals who are not registered healthcare professionals. Adopted from the World Health Organisation classification of disability assistance and support [12], the types of interventions that may be included in our study are community support and independent living; residential support services; respite services; support in education and employment; community access, assistance animals; information and advice services; and other support to caregivers and the assistive devices and technologies. Further details of the types of services and their definitions are outlined in the S1 File.
3. The study focused on people with disability and people with health conditions or impairments that may lead to disability, including sensory/speech (e.g., hearing loss), neurological (e.g., multiple sclerosis), physical (e.g., incomplete use of arm), intellectual (e.g., development delay), cognitive (e.g., dementia), and psychosocial disabilities (e.g., autism) or their caregivers. The categories of disability included in this study are detailed in the S2 File. Although disability is usually defined as having functioning difficulties that last for six months or more [10], previous studies often did not make such a distinction. Therefore, the inclusion criteria were kept broad rather than limited to health conditions that were expected to last six months or more.

### Search strategy

Different electronic databases were searched for studies published between 1 January 2005 and 31 August 2019, and an updated search was conducted to cover studies published between 1 September 2019 and 20 October 2021. The databases included PubMed, AgeLine, Business Source Complete, CINAHL Plus, Communication and Mass Media Complete, EconLit, and SPORTDiscus with Full Text. The reference lists of included studies were hand-searched to identify any further studies that met the inclusion criteria. We excluded studies prior to 2005 because recent studies were more likely to reflect better the current knowledge in the economic evaluation methods and be more relevant for current policy making.

The search strategy was developed based on the inclusion criteria listed above. Medical Subject Headings (MeSH) and keywords were used to indicate the type of economic evaluation, disability and intervention (see S3 and S4 Files).

Search results were managed using Covidence online software. At the importing stage, the software removed duplicates across the databases. Two authors independently screened titles and abstracts for relevance. The third author resolved conflicts if they occurred. The two reviewers then independently assessed the full text of the selected studies. Conflicting decisions were resolved between the two primary reviewers; when consensus could not be reached, the third reviewer acted as an adjudicator.

### Data extraction

A data extraction form was used to extract data on the intervention information (study design and methodology, settings, detailed intervention, and control description) and economic evaluation information (outcomes and QALYs, study perspective in terms of the relevant stakeholders considered, and the cost considered [11, 13], sensitivity analysis, subgroup analysis).

Two authors extracted the key information independently, including the cost items, the type of economic evaluation, perspective, time horizon and sample size. The results of the extraction were then compared. Conflicting decisions were resolved where possible between the two primary reviewers. The third reviewer acted as an adjudicator if conflicts were not resolved.

Information less likely to be subject to differences of opinion (i.e. interventions, countries, age and population) was extracted only by the first author. The second author completed an independent extraction of these items on a 10% random sample. A third reviewer independently compared the results of these two processes. Dual extractions were not performed in these areas because no more than 10% of substantial errors occurred [14].

We assessed the reporting completeness of studies using the 24-item Consolidated Health Economic Evaluation Reporting Standards (CHEERS) checklist [15]. CHEERS was intended to guide rather than assess reporting thus there is no explicit rating scale. The checklist was adapted by the authors to categorise each CHEERS item into: “Complete”, “Partially complete”, “Not complete”, and “Not applicable” to assess the reporting completeness of each CHEERS item–see the S5 File for further details. Two authors carried out the CHEERS reporting completeness assessment independently for all included studies. Conflicts were firstly resolved between the two authors. If no consensus was reached, a third author adjudicated.

## Results

### Scope of the economic evaluations

**Fig 1:**
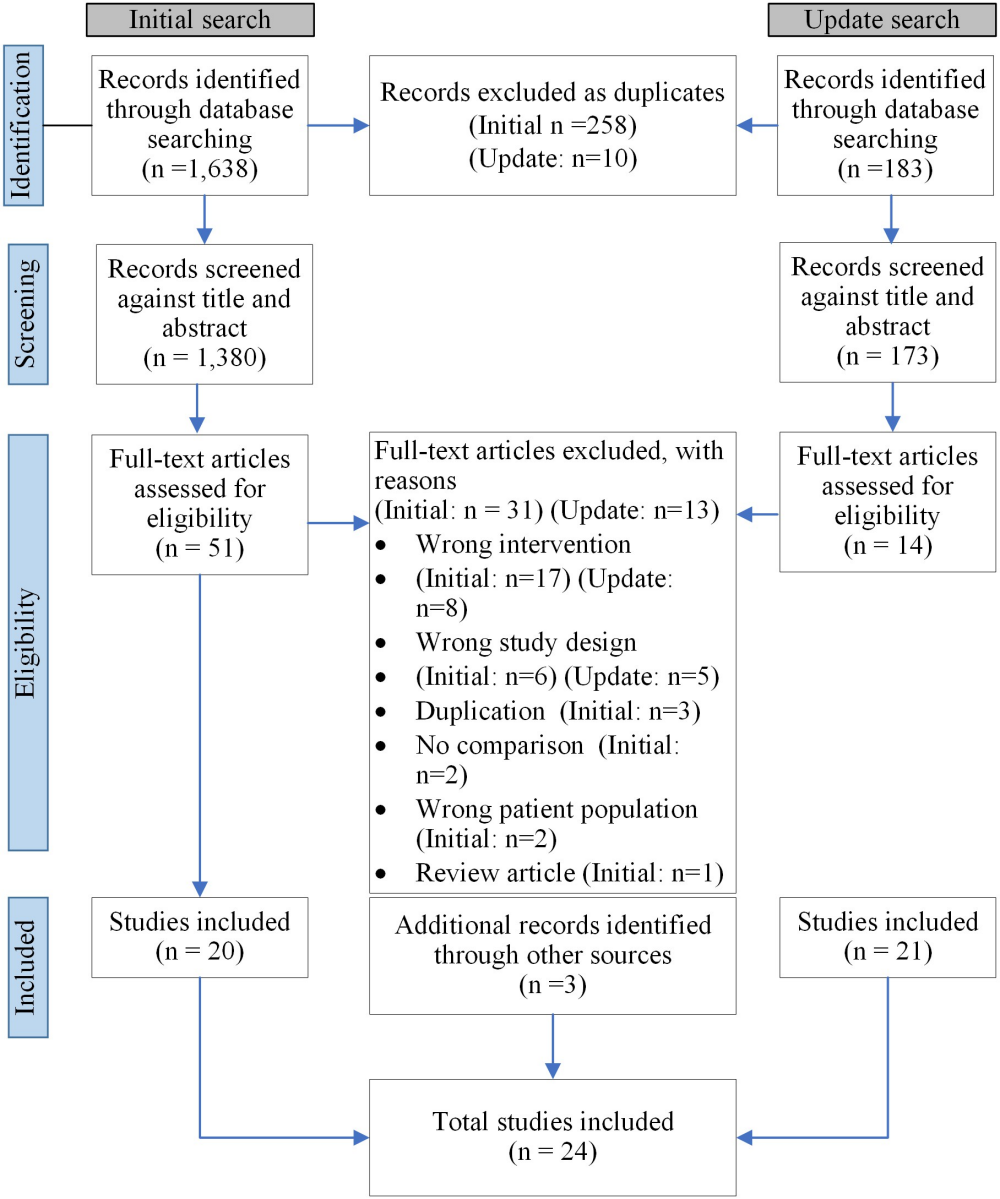
A PRISMA flow chart of studies included and excluded at each stage.

Fig 1 shows a flow diagram of the process applied in study selection. In the initial search, we identified 1,630 articles and selected for inclusion 20 articles. In the updated search covering the period two years after the initial search, we identified 183 articles and selected for inclusion one article. Three articles were added from a hand-search of the references of the included articles. We included 24 articles in the end.

We first describe the scope of the evidence. Among the 24 studies, six intervention areas were identified with 10 focused on employment interventions [16–25], and six on community support and independent living interventions [26–31]. Three studies related to support for caregivers [32–34], two studies on anti-stigma campaigns that may reduce discrimination [35, 36], another two on residential support [37, 38], and one on assistance animals [39].

Of the various types of disabilities, nearly half of the studies focused on interventions targeting people with mental illnesses [16–19, 21, 24, 32, 33, 35, 36]. Four studies were about people with dementia [27, 28, 31, 34], three about people with musculoskeletal disorders [22, 23, 25], and two about people with intellectual disabilities [37, 38]. There was only one study each about stroke [26], cerebral palsy [29], multiple sclerosis [30], vision loss [39] and autism [20]. Further details can be found in S2 Table.

As shown in Table 1, the volume of studies has not increased over time. Six studies were conducted between 2005 and 2009, 10 between 2010 and 2014, and eight between 2015 and 2021. However, the type of economic evaluation conducted did change over time. Even though some health funding bodies recommend cost-utility analysis (in which the outcomes are commonly measured using a single metric that incorporates both quantity and quality of life, i.e, QALYs) for comparability, it has not gained popularity until recently. In the early years of 2005 and 2009, only one study conducted a cost-utility analysis [32]. The other studies were cost-benefit analysis (i.e, converting outcomes to monetary terms) [16, 38, 39], cost-effectiveness analysis (i.e., using natural unit outcomes) [32, 34], or cost-consequences analysis (i.e., reporting outcomes and costs separately) [37]. Between 2010 and 2014, cost-effectiveness analysis became the most popular economic evaluation type that eight out of ten studies published in this period used either cost-effectiveness analysis alone [28, 35] or further alongside with cost-benefit analysis [18, 23, 36] or cost-utility analysis [20, 22, 23, 33]. From 2015 to 2021, cost-utility analysis became the dominating economic evaluation method, and seven out of eight studies used this method either alone [25, 29] or with cost-benefit analysis [19, 21, 26, 27, 31]. We also found that among the 24 studies, three studies only performed cost-utility analysis [25, 29, 30].

**Table 1:** Summary of the key characteristics of the included studies.

Author (Year)	Intervention and Controls	Type of economic evaluation	Perspective	Time horizon (months)	Discount Rate	Primary outcomes Difference (Difference or Intervention vs. Control)	Conclusion by Author
**Employment**							
Chalamat (2005)	Individual placement and support (IPS) vs. Current vocational rehab + open employment services	CBA	Health sector	12	Not mentioned	*Total net benefit of government and* *individual*: Not improved	IPS costs more than monetary benefits, but the evidence base is weak.
Hoffman (2014)	Supported employment program vs. Traditional vocation	CBA	Not mentioned	60	No discount	*Social ROI with mental health treatment (%)*: Improved	Effectiveness of supported employment in improving competitive work outcomes sustained beyond 2 years.
Knapp (2013)	Individual placement and support (IPS) vs. Best typical vocational rehab service in each city followed the train-and-place approach	CEA CBA	Health and social services	18	No discount	*NON-QALYs* *Additional days worked in comparable settings*: Not reported *Additional % of individuals who worked at least 1 day*: Improved	Evidence of cost-effectiveness
Lammerts (2017)	Participatory supportive return to work program vs. Usual care	CEA CUA	Societal Social insurer	12	No discount	*NON-QALYs*: *Duration until sustainable RTW (days)*: Not improved *QALYs*: Not improved	Intervention is not cost-effective in improving sustainable RTW nor in gaining QALYs, and did not yield a positive financial return.
Mavranezouli (2014)	Supported employment program vs. Disability employment advisors service at a job centre	CUA CEA	Main analysis: Unclear Secondary analysis: Health and social services (with accommodation but not medical costs) Health and social services (with medical costs but not accommodation)	96	Discounted costs and outcome at 3.5%	*NON-QALYs*: *The number of weeks in employment*: Improved *QALYs*: Improved	Supported employment is cost-effective compared with standard care.
Saha (2018)	Individual enabling and support models (IES) vs. Traditional vocational rehab services (TVR)	CUA CEA	Societal	12	No discount	*QALYs*: *EQ5D* Not improved *MANSA*: Improved	Intervention is likely cost-saving and potentially cost-effective if measured by MANSA.
Squires (2012)	Workplace + education+ physical activity intervention vs. usual care	CEA CUA	Health and social services Societal Employer	Lifetime	No discount	*NON-QALYs*: *Days of sick leave avoided*: Improved *QALYs*: Improved	Physical +education +work place most effective and cost saving
Sutton (2020)	Individual placement and supported employment vs. Usual care	CUA	Societal	24	No discount	*QALYs*: Not improved	Intervention likely to be effective but not cost-effective
Vermeulen (2013)	Participatory return to work program vs. Usual care	CEA CUA CBA	Social Insurer Societal	12	No discount	*NON-QALYs*: *Duration until sustainable RTW*: Improved *QALYs*: Not improved *CBA*: return on investment: 89%	Evidence of cost effectiveness
Yamaguchi (2017)	Cognitive remediation +Supported employment vs. Traditional vocational services (TVS)	CEA	Health and social services	12	No discount	*NON-QALYs* *Competitive employment*: Improved *Employment tenure*: Improved 0.1 improvement in the *BACS-J*: Improved	CR+SE appeared to be cost-effective compared with TVS.
**Community support and independent living**							
Adie (2017)	Wii (video game) vs. Tailored exercises	CUA CEA	Not mentioned	1.5 (Arm function, primary) 6 (QALYs)	No discount	*NON-QALYs*: *Action research arm test score*: Not improved *QALYs*: Not improved	Intervention NOT superior to comparison.
D’Amico (2015)	12-week walking program +usual care vs. Usual care	CUA CEA	Societal Health and social services	3	No discount	*NON-QALYs* *NPI*: Not improved *ZBI*: Not improved *GHQ*: Not improved *QALYs* (DEMQOL-P) Not improved	Evidence of cost effectiveness focusing on behavioural and psychological symptoms (lower ICER), but weak evidence of it when considering QALYs
Davis (2013)	Resistance training OR Aerobic training vs. Balance and tone (such as Taichi, yoga)	CEA	Health sector	6	No discount	*NON-QALYs* Aerobic VS Balance/tone stroop test: Improved Resistance VS Balance/tone stroop test: Improved	Resistance training and aerobic training are more cost effective than balance and tone classes. Resistance training is promising in altering cognitive decline.
Slaman (2015)	Counselling on daily PA Physical fitness training Counselling about sports participation vs. Usual care	CUA	Societal Health sector	12	No discount	*QALYs* Improved	Interventions might be cost-effective or cost-saving, but the uncertainty of ICER is significant.
Tosh (2014)	12-week exercise intervention +usual care vs. Usual care	CUA	Health sector Societal (Secondary perspective)	9	No discount	*QALYs (EQ-5D)*: Not improved *QALYs (SF-6D)*: Not improved	Intervention is likely to be cost-effective.
Woods (2016)	REMCARE Joint reminiscence groups vs. Usual care	CUA CEA	Health and social services	10	No discount	*QoL-AD*: Not improved *QALYs*: Not improved	Weak evidence of clinical effectiveness or cost-effectiveness of intervention.
**Support to caregivers**							
Charlesworth (2008)	Befriender program (BF) vs. Usual Care	CEA CUA	Societal Health and social services Insurer Individual	15	Discounted costs at 3.5%	*NON-QALYs*: *HADS* Not improved (Caregiver) *QALYs*: Not improved (Caregiver)	Intervention is neither an effective nor cost-effective
Joling (2013)	Family meetings vs. Usual care	CEA CUA	Societal	12	No discount	*QALYs*: Not improved *NON-QALYs*: Not improved	Intervention is not cost-effective when considering reducing burden of informal care.
Nichols (2008)	12 individual educational sessions vs. Two “check-in” phone calls	CEA	Not mentioned	6	No discount	*Non-QALYs* Improved	Intervention was cost-effective in giving spare time for caregivers.
**Anti-Stigma**							
Clement (2012)	Comparison among three interventions: Watch a DVD of service years/informal caregivers talking about their experiences; Watch a similar live presentation; Attend a lecture	CEA	Not mentioned (Included only the intervention costs)	Immediately after the intervention, and 4 months	No discount	Immediate after: *NON-QALYs* Not improved 4 Months: *NON-QALYs* *MICA and SCILO*: DVD vs. lecture: Not improved Live vs. lecture: Not improved *RIBS*: DVD vs. lecture: Improved Live vs. lecture: Not improved	DVD has the best value for money
Evans-Lacko (2013)	Campaign aware (seeing any of ads of a marketing campaign) vs. Not campaign aware	CBA CEA	Not mentioned	Not mentioned	Not mentioned	*NON-QALYs* *MAKS*: Improved *CAMI*: Improved *RIBS*: Improved *ROI (incl*. *service use and employment rates)*: Improved	Intervention is potentially cost effective and of low cost.
**Assistance Animal**							
Wirth (2008)	Guide dog vs. Without guide dog	CBA	Not mentioned (Included cost per guide dog, reduction in formal and informal care)	96	Discounted only costs at 3%	*Non-QALYs* Improved	Costs of dog guides offset only limiting the number of costs, however a major limitation of not including QALYs as the outcome.
**Residential Support**							
Felce (2008)	Semi-independent living vs. Fully staffed group home	CCA	Not mentioned	Unclear (weekly costs)	No discount	Improvement and no improvement in different *NON-QALYs* outcomes	Two living arrangements were strong in different areas, but semi-independent living could offer certain cost-effective lifestyle advantages.
Spreat (2005)	Community placement vs. Institutional placement	CBA	Not mentioned	Unclear	Unclear	NON-QALYs *Healthcare indicators & Health rating*: Not improved *Community integration participation in the community (hour)*: Improved *Family contact scale*: Improved *Service intensity Service (hour/month)*: Improved	Costs are lower in community programs, and also higher levels of services. But institutional programs offered more vocational opportunities.

**Notes**: BACS-J: Brief assessment of cognition in schizophrenia-Japanese; CAMI: Community attitudes towards the mentally ill; CBA: Cost-benefit analysis; CCA: Cost-consequence analysis; CEA: Cost-effectiveness analysis; CUA: Cost-utility analysis; DEMQOL-P: Dementia Quality of Life measure-proxy, a dementia-specific quality of life instrument; GHQ: General health questionnaire; HADS: Hospital Anxiety and Depression Scale; HS: Health sector (perspective); MAKS: Mental health knowledge schedule; MANSA: Manchester Short Assessment of Quality of Life, a quality of life instrument; MICA: Mental illness clinician attitude scale; MINI: Mini International Neuropsychiatric Interview; NPI: Neuropsychiatric Inventory, assessment for neuropsychiatric symptoms; OR: Odds ratio; QALYs: Quality-adjusted life years; ROI: Return of investment; RTW: Return to work; SCILO: Social contact intended learning outcome schedule; ZBI: Zarit caregiver burden inventory, measurement for caregiver burden;

### Methodology assessment

#### Outcomes and outcome measurement

We assessed the variability in how outcomes were measured and whether different outcomes were associated with whether an intervention was likely deemed cost-effective. A comparison of the use of QALYs and non-QALYs outcomes is of particular interest because having a common outcome like QALYs makes it easier to compare the relative cost-effectiveness of interventions across different studies, however, its limitation in being able to capture outcomes beyond health means it may be a less preferable outcome by some studies.

As presented in Table 1, there were more studies using a diversity of non-QALYs outcomes than studies using QALYs. Even for interventions of similar nature like employment programs which mainly compared supported employment versus usual care/services, the outcomes ranged from additional days of work in comparable settings [18], the number of weeks in employment [20] to the odds ratio of competitive employment [24]. These outcomes were selected without consensus or indication of which ones are more appropriate.

Studies using non-QALYs outcomes were more likely to conclude that their intervention was cost-effective compared to studies using QALYs as the outcome. For example, in the employment interventions, four out of the six studies using QALYs found only minor or no change in QALYs [19, 21, 23, 25]. The other two studies [20, 22] using QALYs reported significant changes when the long-term impacts of employment interventions were assessed. Studies using non-QALYs employment-related outcomes found their interventions effective and likely cost-effective [16–18, 24]. In the 24 studies, nine studies used both QALYs and non-QALYs outcomes. Among them, only two showed positive incremental QALYs [20, 22]; however, five reported improved non-QALYs outcomes [20–23, 27]. When each study uses different types of outcomes in their evaluation it becomes difficult for policy makers to compare the relative value for money across interventions and decide which interventions should be prioritised. This is even more important when the choice of outcome to be used in the evaluation can vastly change the overall conclusion on whether or not the intervention is cost effective.

#### Perspectives and costs

Economic evaluations are often conducted from a particular perspective, which defines what cost implications are considered relevant for the funding decision. Some decision makers may want to ignore the cost implications for some payers. For example, if a health sector perspective is adopted, only costs incurred by the health sector are considered. This is important because whether costs are included ore excluded can change the perceived cost effectiveness of the intervention. The cost items and the payers (when stated) are documented in Table 2. The first two columns present the first author of the study and the type of intervention. The area with the dots indicates the cost items included in each study, and the area with the tick indicates the payer. For example, Squire et al. [22] (study #1 in the table) evaluated an employment intervention from the societal perspective. The medical and non-medical cost items were listed with the green dots indicating the cost items that had been included. From the columns to the right, we see that the author considered costs borne by the government and the employer.

**Table 2:** Cost items and payers by perspective.

			Direct medical costs									Direct non-medical costs					Indirect costs				Payers				
	Study/author	Intervention	GP	Hospital admission	Hospital emergency	Medical specialist	Allied health	Rehab/day care	Health professional visits	Image/lab research	Medication	Intervention Costs	Social services	Welfare benefits	Insurance Contributions	Other costs not listed	Productivity costs of patients (in paid work)	productivity costs of patients (non-work related)	Productivity costs of informal Caregivers (in paid work)	productivity costs of informal Caregivers (non-work related)	Government	Employer	Charity	Individual	Unclear
**Societal (n = 9)**																									
1	Squires	E																			√	√			
2	Saha	E																							√
3	Lammerts	E																							√
4	Sutton	E																			√			√	√
4	Tosh	CM																			√		√		
5	Slaman	CM																							√
6	Slaman	CM																							√
7	D’Amico	CM																						√	√
8	Joling	CR																							√
9	Charlesworth	CR																			√		√	√	
**Health and social sectors (n = 8)**																									
10	Mavranezouli	E																			√				
11	Mavranezouli	E																			√				
12	Knapp	E																			√				
13	Yamaguchi	E																			√				
14	Squires	E																			√				
15	D’Amico	CM																							√
16	Woods	CM																			√		√		
17	Charlesworth	CR																			√				
**Health sector (n = 4)**																									
18	Chalamat	E																			√				
19	Davis	CM																			√				
20	Slaman	CM																							√
21	Tosh	CM																			√				
**Social insurer (n = 2)**																									
22	Lammerts	E																			√				√
23	Vermeulen	E																			√				
**Employer (n = 1)**																									
24	Squires	E																				√			
**Voluntary (n = 1)**																									
25	Charlesworth	CR																					√		
**Individual (n = 1)**																									
26	Charlesworth	CR																						√	
**Not mentioned (n = 9)**																									
27	Hoffman	E																			√				√
28	Mavranezouli	E																			√				
29	Adie	CM																							√
30	Nichols	CR																							√
31	Spreat	R																			√				
32	Felce	R																							√
33	Clement	AS																							√
34	Evans-lacko	AS																			√				
35	Wirth	AA																							√
Percentage Included (%)			60	60	43	69	69	43	37	24	54	94	49	3	12	26	35	7	16	8	54	6	9	11	43

Note: E: Employment; CM: Community; CR: Carer; R: Residential; AS: Anti-Stigma; AA: Assistance Animal; GP: General practitioner; Green shade = Included; Red shade = Not included; Yellow shade = Not included because of no impact; Blue shade = Not included because of no data; √ = Included as payer; A study may use more than one perspective.

We firstly assessed whether the studies reported the perspective and what perspectives they adopted. In our study, we found that nine studies did not mention the perspective(s) adopted [17, 20, 26, 34–39]. Among the studies that reported the perspectives, very few reported their perspective to the health sector perspective separately which is the preferred perspective by some government funding bodies. Ten studies adopted the broadest societal perspective [19, 21–23, 25, 27, 29, 30, 32, 33]; eight studies reported taking the health sector and social service perspectives [18, 20, 22, 24, 27, 30, 31], four reported taking the health sector perspective alone [22, 28–30]. Two studies also took the social insurer perspective [19, 23]. One study each also added the employer [22], voluntary [32] and individual [32] perspectives. We found that studies on community support interventions were more likely to adopt a health sector perspective, which does not consider productivity costs.

Among the 10 studies taking the societal perspective, all of them included intervention costs (n = 10), but it was not always clear who the payer was or was likely to be. All 10 studies included general practitioner costs [19, 21–23, 25, 27, 29, 30, 32, 33]; only three studies [25, 27, 30] explicitly stated that hospital emergency costs were included. Productivity costs (related to work) for people with disability were included in seven studies, but only two included productivity costs for caregivers. We can see from Table 2 that government costs were more frequently considered than costs to others, even when the societal perspective was being taken. In only three studies were cost implications for charities, insurers, employers, or individuals considered [22, 25, 32].

Among the studies that adopted the perspective of the health sector and social services (n = 8) or the health sector (n = 4), the medical costs included were highly variable. More than 75% included the GP costs [20, 22, 24, 25, 27–32], hospital in-patient costs [18, 20, 24, 25, 27–31], hospital outpatient costs, [18, 20, 22, 24, 25, 27–32] and allied health costs [18, 20, 22, 24, 25, 27–32]. Only five of the eight studies reported social services costs when taking the perspective of the health sector and social services [18, 20, 24, 27, 31, 32].

Among the six studies with a time horizon longer than a year, only three [20, 32, 39] applied a discount rate to reflect preferences for benefits today rather than in the future.

### Reporting completeness

Information on the percentage and number of items with complete reporting on the CHEERS checklist is provided in the last column of the S1 Table. Eight studies (33%) complete at least 80% of the relevant reporting items [16, 19, 23, 27, 28, 31–33]. In general, the reporting was more complete for studies published more recently and those focusing on either employment, community support or caregiver support interventions.

The key attributes of each CHEERS item and the number of studies that complete, partially complete, do not complete or are not applicable against each item standard are presented in the S2 Table.

## Discussion

This systematic review assessed the quantity and comparability of economic evaluations of social interventions for people with disability. The review of the 24 included studies revealed what is currently lacking in the evidence of the economic evaluations in this field and how to improve the comparison of future studies.

### Findings and discussion

Economic evaluations are widely used for assisting funding decisions. However, we found it challenging to use the evidence included in our systematic review to compare the relative cost-effectiveness of different interventions and thus serve the purpose of assisting funding decisions for social interventions for people with disability. Two main concerns should be noticed.

First, the evidence was limited in number and scope. To allow governments to make evidence-based funding decisions, a broad picture of cost-effectiveness of interventions that cover support to different life aspects and people with different restrictions should be available. However, our findings show that half of the evidence were focused on people with mental illnesses, which was a much higher percentage than the estimated 1 in 8 people with mental disorders globally [40].

We are also aware that some interventions targeting a range of social determinants of health for people with disability (e.g., information and communication, education or physical environment) [41] had not been evaluated. Without evaluating different social interventions for people with disability and then comparing the results, policymakers may not be likely to gain an idea of which areas should be prioritised to be funded when resources are limited.

Second, the existing evidence is highly variable in how both outcomes and costs were measured and thus lacks comparability. For decision makers, it is difficult to decide if they should prioritise an intervention that costs $40,000 per QALY or the one that costs $3,000 per additional day of working. Although cost-utility analysis have gained more popularity in recent years, the widely used health utility instruments primarily focus on physical health and thus are likely to omit the broad benefits that go beyond health [42]. For example, transitioning from institutional to community living may improve not only mental health, but also overall well-being through a greater sense of independence and control over one’s life. There has been heated debate about whether we should use QALYs to quantify health outcomes in disability interventions [43]. One primary concern is that the QALYs calculation may devalue interventions that do not aim to bring back “full health or functioning” to the person but instead support them in achieving high levels of wellbeing despite their current conditions [43]. People with disability may be highly satisfied in reaching a particular functional level instead of paying the additional mental, physical and emotional burden needed to achieve full functioning (or full health). In this case, the interpretation of the cost-utility analysis results that use QALYs for social interventions for people with disability may be misleading.

We found that only two studies included were based solely from the perspective of the health sector. This may be attributable to the fact that the costs of such social interventions are likely spread to sectors other than health. However, for the studies which adopted a wider perspective than health sector, we found that the cost items included were highly heterogeneous. For example, some studies included the productivity costs of informal caregivers in the societal perspective while others did not, which could potentially impact the total amount of the costs as cost to informal caring may be substantial. In most of the cases where some items may be relevant but missing, we are not sure if they were excluded because they had no impact [32, 33] or because there was no data [16]. Therefore, even when studies presented similar results (e.g., $7,000/QALY), it remained unclear whether the interventions had the same value for money.

The above issues indicate that after a decade of Drummond, Weatherly and other authors publishing their concerns on economic evaluations, similar challenges remain in conducting economic evaluations for social interventions for people with disability. Unlike clinical interventions for which QALYs have become the gold-standard measurement for outcomes and relevant costs are often confined to the health sector [44], social interventions often consist of a mixture of different types of services, and there is less consensus on how to measure the outcomes and costs involved consistently. The lack of consistency across the studies included in this review makes it hard for policymakers to compare and draw clear conclusions on the relative cost-effectiveness of the interventions. More economic evaluation studies are required to inform priorities for funding disability interventions; meanwhile, more considerations and guidance are needed to ensure comparability across studies.

### Recommendation for future research

Given the challenges and concerns discussed above, we believe that the research community should endeavour to further develop ways to ensure comparable evidence is available to inform evidence-based policymaking. Recommendations of areas for improvement are highlighted as follows:

Outcome measures: Researchers should justify the choice of measures to be chosen for the calculation of QALYs and non-QALYs outcomes. In the short run, when doing economic evaluations for social interventions for people with disability, researchers should consider using both the QALYs (in cost-utility analysis) for comparisons and non-QALYs outcomes (in cost-effectiveness analysis) if the available outcome measures to calculate QALYs are not likely to capture the benefits of the interventions. Consequently, when policymakers review the evidence, they can both compare the cost-effectiveness across different interventions and be aware of the benefits beyond health. In the long run, consideration should also be given to developing and using a validated disability-specific well-being instrument that could be more sensitive and covers broader outcomes, including important life areas such as independence, safety and sense of belonging with less emphasis being placed on their levels of functioning. In this case, researchers could use this well-being instrument to produce evidence that reflects intervention impact beyond health and is comparable across studies.

Perspective: The health sector perspective is recommended by some government funding bodies for economic evaluation. However, as we found in this study, the costs and benefits of social interventions for people with disability are most likely to go beyond health. As a result, we recommend that researchers consider incorporating both the health sector and social perspectives when doing economic evaluations in this area. The former is the preferred perspective by some government funding bodies, while the latter would capture more costs and impacts. Researchers should also be clear about the likely impacts on costs in sectors other than health so that policymakers could understand the implications of taking a narrow health sector perspective.

Costs: Researchers should make explicit which costs were considered, and why some costs that maybe relevant were not included. Using a comprehensive standardised costing template can help collect costing information, report on costing decisions and justify the costing perspective in a more consistent way. If researchers reported zero costs or stated and justified non-relevant cost items, this would significantly enhance transparency and comparability in cost reporting. Future studies should also consider costs particularly relevant to the disability sector, such as costs of informal caring or those incurred by charities/non-government organisations, to reduce the likelihood of cost-shifting by only considering a government cost perspective.

### Strength and limitations

This study is the first systematic review of the economic evaluations of social interventions for people with disability. It describes the heterogeneity of the methodologies used in the current economic evidence base and how the evidence is distributed across types of disability, intervention areas, and geographic locations. The review was undertaken using a comprehensive search strategy following a registered protocol. While this review provides critical evidence to help inform future research in this field, there are a number of limitations.

First, studies not in English were excluded. This could lead to the potential exclusion of studies, omitting particular relevant studies from non-English countries. Second, while we included broad search terms to capture as many relevant studies as possible, many studies did not report whether health conditions and/or impairments were likely to last more than six months. Therefore, some studies may have included people with an impairment but not disability. However, because the health conditions and impairments mentioned are closely related to disability, the economic evaluations in these studies are still likely to be valuable for decision-making in this field.

Lastly, this review evaluated what evidence was (and was not) considered in an economic evaluation reporting. It did not attempt to evaluate the quality of the evidence used, for example, the potential for bias in the estimated effect sizes. While most of the included economic evaluations were conducted alongside a randomised control trial, and the quality of the trial can also affect the quality of the economic evaluation, evaluating this dimension was out of scope for the current review.

## Conclusion

Economic evaluation evidence is needed to facilitate more evidence-informed decision-making in the disability sector. However, this review found that the current evidence base for social interventions is limited in several ways. In summary, it is limited in scale and scope, inconsistency in methodologies used, especially concerning the measurement of outcomes, selection of cost items and the perspective adopted, all of which can alter the interpretation of the evaluation results. More rigorous economic evidence is needed to support prioritisation decisions. More research is needed to develop a disability-specific outcome measure that could capture broader benefits, providing guidelines on what cost items to include, and adopting a perspective beyond health sector to maximize the usefulness of such evidence. Resources should also be put in to assist equitable and inclusive decision-making. This would also allow the cost effectiveness of alternative interventions to be easily compared.

## Supporting information

S1 File Types of services. (DOCX)

S2 File Types of participants. (DOCX)

S3 File Pubmed search strategies. (DOCX)

S4 File Ebscohost search strategies. (DOCX)

S5 File Description of the CHEERS rating system. (DOCX)

S6 File PRISMA checklist. (DOCX)

S1 Table Characteristics of the interventions and the reporting completeness of studies. (DOCX)

S2 Table Number of studies assessed on reporting quality against the CHEERS guideline. (DOCX)
