# Supplementary material for: Social interventions to support people with disability: A systematic review of economic evaluation studies
Source: PLoS One. 2023 Jan 20;18(1):e0278930. doi: 10.1371/journal.pone.0278930 (PMC9858707; doi:10.1371/journal.pone.0278930)
Supplement: S1 File — (DOCX) [file pone.0278930.s001.docx]

**S1 File: Types of services**

Adapted from the WHO model (World Health Organization, 2011), the following types of services were included in the review:

(1) Community support and independent living: assistance with care and housework, such as personal assistance, home care packages, and home or community physical activity programs

(2) Residential support services: independent living, group living, home modification, or institutional housing

(3) Respite services: temporary breaks for carers and people with disability

(4) Support in education and employment: support in school such as classroom assistance for children in need, supported employment programs

(5) Community access: including day care centres and improvement of physical environments such as public facilities

(6) Assistance animals: such as guide dogs

(7) Information and advice services: such as helpline or information centre, campaigns and advocacy

(8) Other support to carers: supports that help reduce the burden of the carers

(9) Assistive devices and technologies: such as hearing aids

**Reference**

World Health Organization. (2002). Towards a common language for functioning, disability, and health: ICF. The international classification of functioning, disability and health.

World Health Organization. (2011). World report on disability 2011 (9241564180). Retrieved from https://www.who.int/teams/noncommunicable-diseases/sensory-functions-disability-and-rehabilitation/world-report-on-disability
