# Supplementary material for: Social interventions to support people with disability: A systematic review of economic evaluation studies
Source: PLoS One. 2023 Jan 20;18(1):e0278930. doi: 10.1371/journal.pone.0278930 (PMC9858707; doi:10.1371/journal.pone.0278930)
Supplement: S2 File — (DOCX) [file pone.0278930.s002.docx]

**S2 File: Types of participants**

The categories and types of disabilities^[[1]](#footnote-1)^ were

• Sensory/Speech disabilities affecting vision and/or hearing/speech.

• Neurological disabilities affecting a person’s ability to control their movements, such as strokes, epilepsy or multiple sclerosis.

• Physical disabilities affecting mobility and/or a person's ability to use their upper or lower body, including physical limitation due to disfigurement or deformity, incomplete use of arms or fingers, incomplete use of feet or legs, brain injury or cerebral palsy.

• Intellectual disabilities including intellectual and developmental disabilities which can relate to difficulties with thought processes, learning, communicating, remembering information and using it appropriately, making judgements and problem solving,

• Cognitive disabilities affecting a person's thought processes, personality and memory resulting, such as dementia/Alzheimer’s disease.

• Psychosocial disabilities affecting a person’s emotions, thought processes and behaviour, such as mental illnesses or autism.

1. Disability is conceived as an interaction between a health condition(s) of a person and the environment. Many studies conducted before the Convention on the Rights of Persons with Disability (CRPD) used disability more broadly, and collapse disabilities into categories as what we used here. Therefore, disability is an umbrella term for the impairment of body or function, a limitation in activities, or a restriction in participation (World Health Organization, 2002). [↑](#footnote-ref-1)
