# Supplementary material for: Social interventions to support people with disability: A systematic review of economic evaluation studies
Source: PLoS One. 2023 Jan 20;18(1):e0278930. doi: 10.1371/journal.pone.0278930 (PMC9858707; doi:10.1371/journal.pone.0278930)
Supplement: S3 File — (DOCX) [file pone.0278930.s003.docx]

**S3 File: PubMed search strategies**

**General search terms (1, 3, 4, 5 and 6 were applied to all searches)**

**For updated search, 6 (date filter) changed to** (“2019/09/01"[PDat] : "2021/10/20"[PDat])

1. “cost-benefit analysis” [TIAB] NOT Medline [SB] OR “cost-benefit analysis” [MeSH Terms] OR “cost effectiveness” [Text Word] OR “economic evaluation” [Text Word]

2. AND disability types [MeSH Terms]

3. AND (Employment OR return to work* OR rehabilitation OR community health services OR communication OR “safety management” [MeSH Terms] )

+specified interventions if available

4. Not screen* [Title]

5. Not protocol [Title]

6. AND ( "2005/01/01"[PDat] : "2019/8/31"[PDat] ) AND Humans[Mesh] AND English[lang]

**Sensory/speech**

- ***Vision loss***

2. AND Blindness [MeSH Terms]

3. AND (Housing OR Employment OR return to work* OR community health services OR communication OR safety management OR sensory aids [MeSH Terms])) OR ("guide dog*" OR "dog guides"[All Fields] OR "assistance animal*"[TIAB]

- ***Hearing loss***

2. Hearing Loss [Mesh Term]

3. AND (Housing OR Employment OR return to work* OR community health services OR communication OR safety management OR sign language* OR Hearing aid OR Ear mold* Or sensory aid [MeSH Terms]

- ***Speaking problems***

2. Phonation Disorder OR Dysphonia [Mesh Term]

3. AND (Housing OR Employment OR return to work* OR community health services OR communication OR safety management OR sign language* [MeSH Terms]

**Neurological**

- ***Stroke***

2. Cerebrovascular Disease OR stroke OR Cerebrovascular accident [Mesh Term]

- ***Epilepsy***

2. Epilepsies &Seizure Disorder [Mesh Term]

3. AND (Housing OR Employment OR return to work* OR community health services OR communication OR “safety management” [MeSH Terms])

- ***Multiple sclerosis***

2. MS (Multiple Sclerosis) [Mesh Term]

3. AND (Housing OR Employment OR return to work* OR community health services OR communication OR “safety management” OR self-help groups OR support groups [MeSH Terms])

**Physical**

- ***Common physical limitation***

2. Physical restraints OR immobilization Or Experimental Hypokinesia [Mesh Term]

3. AND (Housing OR Employment OR return to work* OR community health services OR communication OR “safety management” OR self-help groups OR support groups [MeSH Terms])

- ***Brain injury***

2. AND brain injur* [MeSH Terms]

- ***Cerebral palsy***

2. AND CP (Cerebral Palsy) [Mesh Term]

3. AND (Housing OR Employment OR return to work* OR community health services OR communication OR “safety management” OR self-help groups OR support groups [MeSH Terms])

**Intellectual**

- ***Development delay/mental retardation***

2. Intellectual disability OR Mental retardation OR intellectual development disorder OR mental deficiency OR Idiocy [MeSH Terms]

3. AND (Housing OR Employment OR return to work* OR communityhealth services OR communication OR “safety management” OR self-help groups OR support groups OR deinstitutionalzation[MeSH Terms])

**Cognitive**

- ***Memory loss***

2. Memory loss OR memory disorder OR Cognitive retention disorders [Mesh Term]

3. AND (Housing OR Employment OR return to work* OR community health services OR communication OR safety management [MeSH Terms]

- ***Dementia/Alzheimer’s disease***

2. Dementias OR Amentia OR Alzheimer’s Disease [Mesh Term]

3. AND (Housing OR Employment OR return to work* OR community health services OR communication OR safety management OR behaviour therapy OR exercise*[MeSH Terms]

**Psychosocial**

- ***Autism***

2. Autism OR Autistic Disorder [Mesh Term]

3. AND (Housing OR Employment OR return to work* OR community health services OR communication OR “safety management” OR self-help groups OR support groups [MeSH Terms])

- ***Mental illness***

2. Mental disorder OR Schizophrenias OR Schizophrenic Disorders OR Bipolar Disorder OR Bipolar Affective Psychosis OR Depressive disorders OR Depressive Syndrome OR Anxiety disorder OR Obsessive-compulsive
