# Supplementary material for: Social interventions to support people with disability: A systematic review of economic evaluation studies
Source: PLoS One. 2023 Jan 20;18(1):e0278930. doi: 10.1371/journal.pone.0278930 (PMC9858707; doi:10.1371/journal.pone.0278930)
Supplement: S4 File — (DOCX) [file pone.0278930.s004.docx]

**S4 File: Ebscohost search strategies**

**General search terms (1, 2, 5 were applied to all searches)**

**For updated search, Date filter changed to** : Date: 1/9/2019 to 20/10/2021

1. economic evaluation OR cost N2 effective* OR cost N2 utility Or cost N2 benefit OR cost N2 consequence*

2. AND “community health” OR Outpatient OR home OR community OR resident* OR home OR “social support” OR “long-term care” OR “informal care” OR social

3. AND disability types

4. AND intervention types

5. NOT screen* or protocol [Title]

Filter: Date: 1/1/2005 to 31/08/2019; Language: English

**Sensory/speech**

- ***Vision loss***

3. AND (blindness or visual impairment or vision loss)

4. AND (Housing OR Employment OR jobs or career or return to work* OR community health services OR communication OR safety management OR sensory aids OR “guide dog*” OR “assistance animals”)

- ***Hearing loss***

3. AND (hearing loss or deafness or hearing impairment or deaf or hard of hearing)

4. AND (Housing OR Employment OR return to work* OR rehabilitation OR community health services OR communication OR safety management OR sign language* OR Hearing aid OR Ear mould* Or sensory aid)

- ***Speaking problems***

3. AND (speech disabilities or speech disorder or Phonation Disorder OR Dysphonia)

4. AND (Housing OR Employment OR jobs or career or return to work* OR community health services OR communication OR safety management OR sensory aids OR sign language*

**Neurological**

- ***Cerebrovascular disease***

3. AND (Cerebrovascular Disease OR stroke OR Cerebrovascular accident)

4. AND (Housing OR Employment OR return to work* or job or career OR rehabilitation OR community health services OR communication OR “safety management”)

- ***Epilepsy***

3. AND (epilepsy or seizures or epileptic)

4. AND (Housing OR Employment OR jobs or career or return to work* OR community health services OR communication OR safety management

- ***Multiple sclerosis***

3. AND (Multiple Sclerosis)

3. AND (Housing OR Employment OR jobs or career or return to work* OR rehabilitation OR community health services OR communication OR safety management OR self-help groups OR support groups)

**Physical**

- ***Common physical limitation***

2. AND (Physical restraints OR immobilization Or Experimental Hypokinesia)

3. AND (Housing OR Employment OR jobs or career or return to work* OR rehabilitation OR community health services OR communication OR safety management OR self-help groups OR support groups

- ***Brain injury***

3. AND (brain injury or head injury or traumatic brain injury or acquired brain injury)

4. AND (Housing OR Employment OR jobs or career or return to work* OR rehabilitation OR community health services OR communication OR safety management OR self-help groups OR support groups)

- ***Cerebral palsy***

2. AND (Cerebral Palsy)

3. AND (Housing OR Employment OR jobs or career or return to work* OR rehabilitation OR community health services OR communication OR safety management OR self-help groups OR support groups)

**Intellectual**

- ***Development delay/mental retardation***

3. AND (mental retardation or intellectual disabilities or developmental disabilities)

4. AND (Housing OR Employment OR jobs or career or return to work* OR rehabilitation OR community health services OR communication OR “safety management” OR self-help groups OR support groups)

**Cognitive**

- ***Memory loss***

3. AND (Memory loss OR memory disorder OR Cognitive retention disorders or memory errors)

4. AND (Housing OR Employment OR jobs or career or return to work* OR rehabilitation OR community health services OR communication OR safety management)

- ***Dementia/Alzheimer’s disease***

3. AND (dementia or alzheimers or cognitive impairment)

3. AND (Housing OR Employment OR jobs or career or return to work* OR rehabilitation OR community health services OR communication OR safety management)

**Psychosocial**

- ***Autism***

3. AND (autism or asd or autism spectrum disorder)

3. AND (Housing OR Employment OR jobs or career or return to work* OR rehabilitation OR community health services OR communication OR “safety management” OR self-help groups OR support groups)

- ***Mental illness***

3. AND (mental health or mental illness or mental disorder or psychiatric illness OR Schizophrenias OR Schizophrenic Disorders OR Bipolar Disorder OR Bipolar Affective Psychosis OR Depressive disorders OR Depressive Syndrome OR Anxiety disorder OR Obsessive-compulsive disorder)

4. AND (Housing OR Employment OR jobs OR career OR return to work* OR community health services OR communication OR safety management)
