# Supplementary material for: Social interventions to support people with disability: A systematic review of economic evaluation studies
Source: PLoS One. 2023 Jan 20;18(1):e0278930. doi: 10.1371/journal.pone.0278930 (PMC9858707; doi:10.1371/journal.pone.0278930)
Supplement: S5 File — (DOCX) [file pone.0278930.s005.docx]

**S5 File: Description of the CHEERS rating system**

To understand how complete studies were, use defined four categories: "Complete", "Partially complete", "Not complete", and "Not applicable". “Complete” means that the authors were able to report and justified the content of the item as required by their study design. “Partially complete” is defined as (1) Studies that fully reported the content but did not justify why the methods were appropriate; and (2) Studies partially reported the contents or justified only part of their parameters (or absence of parameters). “Not complete” is defined as missing content of the item without justification. The "Not applicable" category applies only to four items:

- measurement and valuation of preference-based outcomes (not applicable if using non-preference-based outcomes) (Item 12 in Table S2);
- choice of model (not applicable if not model-based) (Item 15 in Table S2);
- the assumptions (not applicable if not model-based) (Item 16 in Table S2); and
- characterizing heterogeneity (not applicable if subgroup analysis not needed) (Item 21 in Table S2).
