# Supplementary material for: Social interventions to support people with disability: A systematic review of economic evaluation studies
Source: PLoS One. 2023 Jan 20;18(1):e0278930. doi: 10.1371/journal.pone.0278930 (PMC9858707; doi:10.1371/journal.pone.0278930)
Supplement: S1 Table — (DOCX) [file pone.0278930.s007.docx]

**S1 Table:** **Characteristics of the interventions, costs, incremental cost-effectiveness ratio and the reporting completeness of studies**

| **Lead Author (Year)** | **Country** | **Type of disability (population)** | | **Type of study** | **Sample size (for analysis)** | | **Age group*** | **Cost** | **ICER**  **(if applicable)** | **Percentage of fully complete on**  **CHEERS checklist**  **(number of**  **S*/ non-**  **NA* items)** | | |
| --- | --- | --- | --- | --- | --- | --- | --- | --- | --- | --- | --- | --- |
|  |  |  |  | | | **Employment** | | | | |  |  |
| Chalamat (2005) | Australia | Psychosocial-  Mental illness  (People with disability) | | Model:  Decision Tree | 7,800 | | Working age | $AUD 10.3M | NA | 77% (17/22) | | |
| Hoffman  (2014) | Switzerland | Psychosocial-  Mental illness (People with disability) | | RCT | Intervention: 39 Control: 49  Total: 88 | | Working age | -CHF 292 / client | NA | 50% (10/20) | | |
| Knapp  (2013) | Six Euro Countries | Psychosocial -Mental illness (People with disability) | | RCT | Intervention: 156 Control: 156  Total: 312 | | Unclear | -£4,022  (-£4,791 to  -£3,239) | ICER for both outcome 1) and 2): IPS dominates CBA: £17,005 in favour of IPS | 71% (15/21) | | |
| Lammerts (2017) | Netherlands | Psychosocial-Mental illness  (People with disability) | | RCT | Intervention: 94 Control: 92  Total: 186  (Imputation) | | Unclear | Societal:  Intervention: £ 106,401  (SD=55,448) Control:  £ 106,693  (SD=60,842) | *(1) RTW:*  -£487/day  (Dominant) *(2) QALY:*  -£125,357/QALY  (Dominated) | 86% (18/21) | | |
| Mavranezouli (2014) | UK | Psychosocial –  Autism (People with disability) | | Model: Decision Tree and Markov Model | NA | | Unclear | Health and social services: £602 | *(1) Employment*  £18/ extra week of employment *(2) QALY*  £5600 /QALY | 70% (16/23) | | |
| Saha  (2018) | Sweden | Psychosocial-Mental illness  (People with disability) | | RCT  Synthesised evidence for  costs | Intervention: 31 Control: 24  Total: 55 | | Unclear | Intervention:  €528  Control:  €7,775 | Not reported for QALY as no change.  No ICER reported for the MANSA outcome despite improvement. | 71% (15/21) | | |
| Squires  (2012) | UK | Physical-Musculoskeletal disorders (People with disability) | | Model:  Markov  Model | NA | | Working age (up to 66 years) | Varied, depending on perspectives. Usual care: £216 | Health and societal perspective (similar)  *(1) QALY*  Physical +education  : £2800/QALY Other two interventions: interventions dominant (lower costs)  *(2) Sick leave:* Workplace: 34 pence/day on sick leave avoided | 74% (17/23) | | |
| Sutton  (2020) | US | Physical- Musculoskeletal disorders (People with disability) | | Observational study for outcome  Synthesised evidence for costs | Intervention: 213 Control: 76  Total: 289 | | Unclear | Linear model:  Intervention: $136,927  Control:  $173, 657  Cost difference not significantly different | Dominated (lower costs more QALYs) –  Net monetary benefit at WTP=$50,000 equal to -$7686  at WTP=$100,000  equal to -$1646 | 47% (7/19) | | |
| Vermeulen (2013) | Netherlands | Physical-Musculoskeletal disorders (People with disability) | | RCT | Intervention: 79 Control: 84  Total: 163 | | Working age | Social insurer:  €2,327  (51 to 4,465) | (1) *ICER (social insurer):*  -€76/day RTW early  (2) *ICER (societal):*  €46,540/QALY | 95% (20/21) | | |
| Yamaguchi (2017) | Japan | Psychosocial-Mental illness  (People with disability) | | RCT | Intervention: 45 Control: 47  Total: 92 | | Working age | -$1,640  (-5,559 to 2,279) | Intervention dominant  *(1) Employment rate*:  -$29/%  *(2) Employment tenure:*  -$23/day  *(3) 0.1 improvement in BACS-J:*  -$387 | 76% (16/21) | | |
| **Community support and independent living** | | | | | | | | | | | | |
| Adie  (2017) | UK | Neurological-Stroke (People with disability) | | RCT | Intervention: 117 Control:118  Total:235 | | Working age; Senior | Intervention:  £1,106 (SD=1656)  Control  £730 (SD=829) | Only reported dominated | 52% (11/21) | | |
| D'Amico  (2015) | UK | Cognitive-Dementia/Alzheimer's disease  (People with disability and carer) | | RCT | 113 dyad | | Unclear | HS:  All negative Societal:  *(1) NPI:* £1,686.4  (-1,407.1 to 4,780)  *(2) ZBI:* £1,641.1  (-1,497.8 to 4,780)  *(3) DEMQOL-P:*  £1,635.9  (-1,520.9 to 4,792.6)  *(4) QALY:* £,1656.8  (-1,592.6 to 4,724.2)  *(5) GHQ:*  £1,657.3  (-1,471.8 to 4,786.4) | HS:  All intervention dominant Societal:   1. *NPI:* £421 2. *ZBI:* £1,055 3. *DEMQOL-P:*   £580   1. *QALY:*   £286 440  *GHQ:* £392 | 86% (18/21) | | |
| Davis  (2013) | Canada | Cognitive-Dementia/Alzheimer’s disease  (People with disability) | | RCT  (3 arm) | Intervention (Resistance) :28  Intervention (Aerobic): 30  Control (BAT): 28  Total: 86 | | Senior | Aerobic:  -CAN$316 Resistance:  -CAN$33 | Both dominant | 80% (16/20) | | |
| Slaman  (2015) | Netherlands | Physical-cerebral palsy  (People with disability) | | RCT | Intervention: 20 Control: 20  Total: 40 | | Working age | Societal:  -€310  HS:  -€265 | Societal:  -€23,664 /QALY  HS:  -€20,229 /QALY | 67% (14/21) | | |
| Tosh  (2014) | UK | Neurological -Multiple Sclerosis (People with disability) | | RCT | Intervention: 60 Control: 60  Total: 120 | | Unclear | £466 (-273 to  1,310) | *(1) QALY (EQ-5D):* HS:  £10,137/QALY  Societal:  £24,897 /QALY *(2) QALY (SF-6D):* HS:  £19,783/QALY | 73% (16/22) | | |
| Woods  (2016) | UK | Cognitive-Dementia/Alzheimer's disease (People with disability and Carer) | | RCT | 350 dyads | | Unclear | *(1) QoL-AD:* £1,544 (Patient)  *(2) QALY:*  £1,544 (Patient)  £1,136 (Carer) | *(1) QoL-AD:*  £2,586 (-20,280 to  24,340) (Patient)  *(2) QALY:* Dominated | 86% (19/22) | | |
| **Support to carers** | | | | | | | | | | | | |
| Charlesworth (2008) | UK | Psychosocial-Mental illness  (Carer) | | RCT | Intervention: 93 Control: 104  Total: 197 | | Working age; Senior | Societal:  £1,813  (-£11,312 to £ 14,984) | *QALY:*  £105,954/QALY | 86% (18/21) | | |
| Joling  (2013) | Netherlands | Psychosocial-Mental illness  (People with disability and Carer of people with dementia, dyad) | | RCT | Intervention: 96 Control: 96  Total:192 | | Unclear | Carer: -€788  Patient: €4,936  Dyad: € 4,149 | 1. *QALY*   -€32,254 (Carer)  €2,574,938  (Patient)  €157,534 (Dyad)     1. *MINI*   -€59011 (Carer) | 86% (19/22) | | |
| Nichols  (2008) | US | Cognitive-Dementia/  Alzheimer's disease  (Carer) | | RCT | 46 dyads | | Working age; Senior | $1160 /person | $4.96/day for an extra hour of non-caregiving time per day for each caregiver | 45% (9/20) | | |
| **Anti-Stigma** | | | | | | | | | | | | |
| Clement  (2012) | UK | Psychosocial-Mental illness  (Public) | | RCT (3 arms) | DVD: 63  Live presentation: 72  Lecture:58  Total: 193 | | Working age | DVD vs. live:   - £575   DVD vs. lecture:  £99 | DVD dominant | 53% (10/19) | | |
| Evans-Lacko (2013) | UK | Psychosocial-Mental illness  (Public) | | Decision Tree | 6,000 | | Working age | *Cost of program:*  £20 million *Cost of conditions:* £48.6 billion | NA | 50% (10/20) | | |
| **Assistance Animal** | | | | | | | | | | | | |
| Wirth  (2008) | US | Sensory/Speech-  Vision loss  (People with disability) | | Synthesised evidence | NA | | Unclear | $40598 (working dog life time, approx. 8 years) | NA | 64% (14/22) | | |
| **Residential support** | | | | | | | | | | | | |
| Felce  (2008) | UK | Intellectual-Intellectual disabilities (People with disability) | | Survey | Semi: 35  Full: 35  Total: 70 | | Working age; Senior | Semi vs Full:  542.1 (SD=348.2)  vs. 1539.0  (SD=639.2) | NA | 50% (10/20) | | |
| Spreat  (2005) | US | Intellectual- Intellectual disabilities (People with disability) | | Survey | Community: 174  Institute: 174  Total :348 | | Unclear | Intervention:  $123,384  Control:  $138,720 | NA | 40% (8/20) | | |
