# Supplementary material for: Social interventions to support people with disability: A systematic review of economic evaluation studies
Source: PLoS One. 2023 Jan 20;18(1):e0278930. doi: 10.1371/journal.pone.0278930 (PMC9858707; doi:10.1371/journal.pone.0278930)
Supplement: S2 Table — (DOCX) [file pone.0278930.s008.docx]

**S2 Table:** **Number of studies** **assessed on reporting completeness against the CHEERS guideline**

| CHEERS  Guideline  Items  Quality (n=24) | **C** | **PC** | **NC** | **NA** | **Key Attributes** |
| --- | --- | --- | --- | --- | --- |
| **Title and abstract** |  |  |  |  |  |
| 1. Title | 1 | 22 | 1 | 0 | Must identify economic evaluation, intervention and comparison |
| 2. Abstract | 21 | 3 | 0 | 0 | Structured summary of studies including objectives, setting, methods, results and conclusions. |
| **Introduction** |  |  |  |  |  |
| 3. Background and objectives | 23 | 1 | 0 | 0 | Explain relevance to decision making |
| **Methods** |  |  |  |  |  |
| 4. Target population and subgroups | 22 | 2 | 0 | 0 | Describe and justify based-case population and subgroups |
| 5. Setting and location | 22 | 2 | 0 | 0 | Location(s) of study and aspects of systems where decisions are made |
| 6. Study perspective | 14 | 2 | 8 | 0 | Describe perspective and relate to costs |
| 7. Comparators | 17 | 6 | 1 | 0 | Describe comparators. If used ‘usual care’, explain what it is |
| 8. Time horizon | 5 | 15 | 4 | 0 | State and justify time horizon |
| 9. Discount rate | 8 | 5 | 11 | 0 | Report the discount rate if applicable. If shorter than 12 months, state that discount is not necessary. |
| 10. Choice of health outcomes | 14 | 10 | 0 | 0 | Report and justify the outcomes used. |
| 11a. Measurement of effectiveness (S)  11b. Measurement of effectiveness (M) | 16  2 | 4  2 | 0  0 | 0  0 | Describe and justify study features |
| 12. Measurement and valuation  of preference-based outcomes | 13 | 0 | 0 | 11 | Applicable only if preference-based outcomes were used |
| 13a. Estimating resources and costs (S)  13b. Estimating resources and costs (M) | 13  3 | 5  3 | 0  0 | 0  0 | Describe methods to estimate costs including the unit costs, and the valuing of costs. |
| 14. Currency, price date, and conversion | 16 | 7 | 1 | 0 | Currency, price date, and conversion |
| 15. Choice of model | 1 | 3 | 0 | 20 | Applicable only if using model to analyse |
| 16. Assumptions | 3 | 1 | 0 | 20 | Applicable only if using model to analyse |
| 17. Analytical methods | 17 | 6 | 1 | 0 | Describe analytic methods such as dealing with missing or skewed data. |
| **Results** |  |  |  |  |  |
| 18. Study parameters | 15 | 8 | 1 | 0 | Report ranges or distributions for parameters such as unit costs, or probabilities in decision tree. |
| 19. Incremental costs and outcomes | 16 | 6 | 2 | 0 | Report difference of mean costs and benefits. Report ICER if applicable. |
| 20a. Characterising uncertainty (S)  20b. Characterising uncertainty (M) | 13  5 | 6  0 | 0  0 | 0  0 | Describe uncertainties related to sampling or other methodological assumptions. |
| 21. Characterising heterogeneity | 5 | 0 | 0 | 19 | Applicable if doing subgroup analysis |
| **Discussion** |  |  |  |  |  |
| 22. Study findings, limitations, generalizability, and current knowledge | 23 | 1 | 0 | 0 | Discuss these aspects |
| 23.Source of funding | 21 | 0 | 3 | 0 | State sources of funding |
| 24. Conflicts of interest | 16 | 1 | 7 | 0 | State conflicts of interests |

Note: C: Complete; PC: Partially complete; NC: Not complete; NA: Non-applicable. Key attributes summarised from the CHEERS checklist
